# Clinical Prediction Scoring Scheme for 24 h Mortality in Major Traumatic Adult Patients

**DOI:** 10.3390/healthcare10030577

**Published:** 2022-03-20

**Authors:** Waratsuda Samuthtai, Jayanton Patumanond, Pawitrabhorn Samutrtai, Thammanard Charernboon, Kijja Jearwattanakanok, Jiraporn Khorana

**Affiliations:** 1 Department of Emergency Medicine, Nakornping Hospital, Chiang Mai 50180, Thailand; waratsamuth@gmail.com; 2 Division of Clinical Epidemiology and Clinical Statistics, Faculty of Medicine, Thammasat University, Bangkok 12120, Thailand; jpatumanond@gmail.com; 3 Department of Pharmaceutical Sciences, Faculty of Pharmacy, Chiang Mai University, Chiang Mai 50200, Thailand; pawitrabhorn.s@cmu.ac.th; 4 Department of Psychiatry, Faculty of Medicine, Thammasat University, Bangkok 12120, Thailand; dr.thammanard@gmail.com; 5 Department of Surgery, Nakornping Hospital, Chiang Mai 50180, Thailand; jkijja@gmail.com; 6 Department of Surgery, Faculty of Medicine, Chiang Mai University, Chiang Mai 50200, Thailand; 7 Center of Clinical Epidemiology and Clinical Statistics, Faculty of Medicine, Chiang Mai University, Chiang Mai 50200, Thailand; 8 Clinical Surgical Research Center, Department of Surgery, Faculty of Medicine, Chiang Mai University, Chiang Mai 50200, Thailand

**Keywords:** multiple trauma, prognostic factor, pre-clinical scoring, early mortality

## Abstract

A death rate of approximately 32.7 in 100,000 traffic injury victims was reported in Thailand. The prediction of early death would identify and enable prioritization of the most severe patients for resuscitation and consequently reduce the number of deaths. This study aimed to develop a clinical prediction scoring system for 24 h mortality in adult major trauma patients. Retrospective-prognostic clinical prediction was applied in the case of 3173 adult trauma patients who were classified into three groups: death within 8 h, death between 8 and 24 h, and alive at 24 h. The predictors were obtained by univariable and multivariable logistic regression, and the coefficient of parameters was converted to predict early death. The numbers of patients who died within 8 h and between 8 and 24 h were 46 (1.5%) and 123 (3.8%), respectively. The predictors included systolic blood pressure <90 mmHg, heart rate ≥120 bpm, Glasgow coma scale ≤8, traffic injury, and assault injury. The scores of 4 indicated a mortality rate of 12% with a high specificity of 0.89. The suggested TERMINAL-24 scoring system can be used for the prediction of early death in the Emergency Department. However, its discrimination ability and precision should be validated before practical use.

## 1. Introduction

In the latest WHO report in 2016, Thailand ranked ninth globally as regards road accidental deaths with a fatality rate of 32.7 per 100,000 [1]. From the Information Technology for Emergency Medical System (ITEM), the number of trauma patients in Chiang Mai, Thailand in 2015–2017 was shown to be approximately 18,000 persons per year, with 270 deaths [2]; therefore, physicians have encountered many injured patients in the Emergency Department. The database of a tertiary hospital in Chiang Mai indicated the average number of trauma patients in the Emergency Department during 2014–2019 to be roughly 14,000 persons per year; 4000 were admitted and 15 died. Given the large number of trauma cases, in addition to the increased number of traffic trauma patients in the past 6 years, using a derivative scoring scheme to predict early death within the first 24 h would be helpful to advocate emergency surgery, prioritize treatment in mass casualty incidents, and inform the relatives of the victims of the chance of death. In situations in which the Emergency Department is crowded and there are many trauma patients at the same time, patients needing surgery need to be prioritized. In addition, if multiple trauma incidents are referred to deficient facilities, efficient transfer of the most severe patients to the trauma center is crucial. In these situations, we need some simple trauma scores to facilitate the rapid triage of multiple trauma patients.

Previous scoring systems for the prediction of death in traumatic patients have been proposed including RTS [3,4], ISS [5], TRISS [6], EMTRAS [7], REMS [8], MGAP [9], and GAP [10], as well as the predictive studies focusing on time and causes of death [11,12,13,14,15,16,17,18,19]. Most systems defined early death as death within 24 h [11,12,17,18,19]. Our study had a focus on earlier death, death between 8 and 24 h, and specifically death within 8 h, which is the period targeting the resuscitation of the patients in an emergency room.

This tertiary hospital in Chiang Mai has been adopting the existing trauma scores such as RTS and ISS, but these scoring systems exhibited low capability of predicting 24 h mortality with the area under the receiver operating characteristic (ROC) curves of 59% and 58%, respectively. The reason for this poor competency may be due to the lack of some predictors, for example, the mechanisms of injury or transfer processes. Therefore, we decided to derive a new scoring system in our setting.

In this study, we aimed to develop a new scoring system to more powerfully identify the prediction of early death in traffic trauma patients, which would be practical in an emergency setting and inform and promote the treatment decision plan for those patients.

## 2. Materials and Methods

All adult traumatic patients aged ≥15 years, categorized as resuscitation and emergency level, who visited the emergency department in this tertiary hospital in the northern region of Thailand between January 2012 and December 2017 were included in this study.

We retrieved medical records from a database called Injury Surveillance system (IS data), which classified trauma patients into 3 groups: died within 8 h, died between 8 and 24 h, and alive at 24 h. These were used as the outcomes of interest. The ATLS program (Advanced Trauma Life Support) developed by the American College of Surgeons, proposes trimodal death as immediate, early, and late death. Our study focused on early death, defined as death within 8 h, and death between 8 and 24 h [20].

Indicator parameters, including the relevant information of the patients, were (1) demographic profiles: sex and age; (2) prehospital profiles: blunt or penetrating injuries, traffic, assault, or falling injury; transfer by referring or an emergency response team; (3) hemodynamic profiles: blood pressure, pulse rate, respiratory rate at triage area, and level of consciousness using Glasgow Coma Scale (GCS). Records with missing data were excluded from the analysis.

To derive the clinical scoring, the parameters across 3 groups were compared by order using a nonparametric test for trend since the process of death is a continuation ratio. The continuous data were analyzed by statistics and clinical judgment, i.e., age ≥ 50, systolic blood pressure < 90 mmHg, pulse rate ≥ 120 bpm, respiratory rate < 10 or >30, GCS ≤ 8, to produce 2 ordinal variables. The predictability of each parameter was explored using a univariable ordinal continuation ratio model, a model based on the continuation ratio of the outcome ordered by the time of death in 3 levels of severity: dead within 8 h, dead between 8 and 24 h, and alive at 24 h. Multivariable ordinal continuation ratio logistic regression was used to evaluate the predictability of combined parameters: odds ratio, coefficient, and *p*-value of significant predictors. The insignificant parameters were individually removed from the model judged by the odds ratio of less than 1 and a *p*-value > 0.05 until all parameters in the model were significant. The coefficients of each significant parameter were converted to item scores with the lowest coefficient as the smallest common division. Each item score was added up, and the total score of each patient was calculated. We called the new scoring system “TERMINAL-24” (Traumatic Emergency Room Major INjury death At Least 24 h).

To test the score performance, a distributive box plot was created to find a score difference and the distribution across levels. Individual score prediction was made by the probability of death in each patient, then the cumulative probability of TERMINAL-24 score was built to indicate a cutoff point to classify the group of patients. The goodness of fit of this model was calibrated using the Hosmer–Lemeshow test from the aspects of the area under ROC and the risk curve of each time band of death. Finally, the mortality rate of each patient according to the TERMINAL-24 score was calculated to validate risk estimation. The internal validity of the model was conducted using a bootstrapping method. We used 3000 bootstrap samples for model derivation and tested the overfitting by the optimism of real performance and model performance.

## 3. Results

### 3.1. Characteristics of Study Subjects

**Figure 1:**
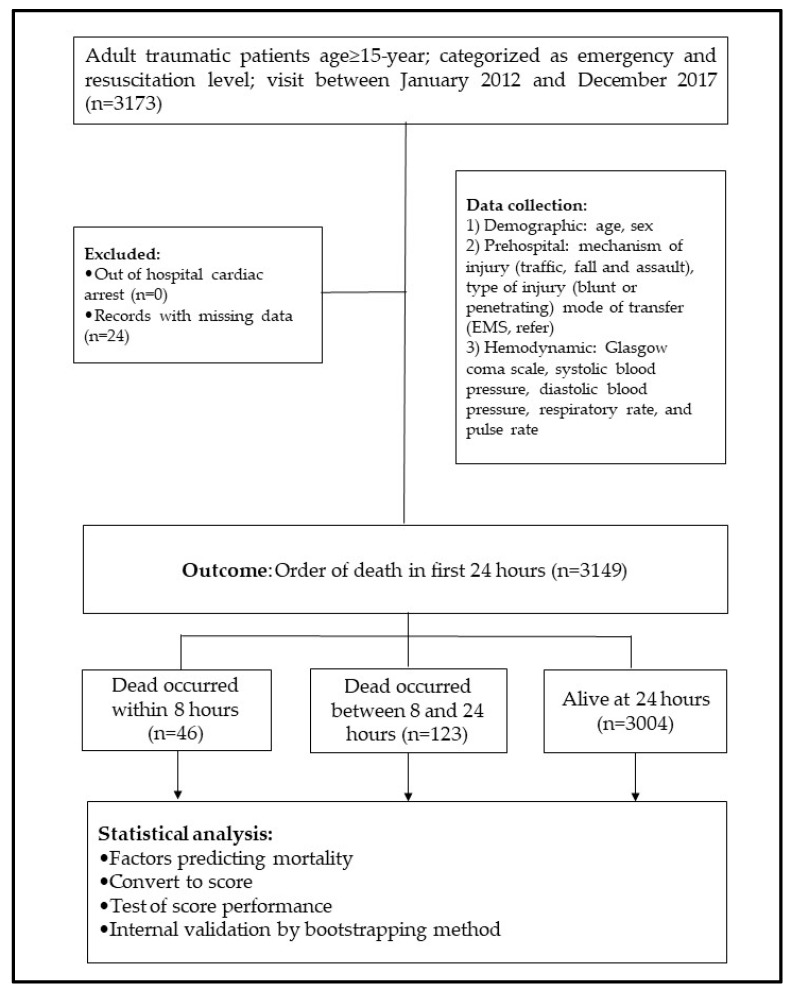
Flow diagram to show the development of TERMINAL-24 score.

A total of 3173 traumatic patients were included in the study, 46 (1.5%) of which were dead within 8 h, 123 (3.8%) died between 8 and 24 h, and 3004 (94.7%) were alive at 24 h, as illustrated in Figure 1. According to the univariable ordinal continuation ratio logistic analysis, the parameters affecting the death order of traumatic patients were hemodynamic profile and traffic injury, as shown in Table 1.

### 3.2. Main Results

As indicated by the multivariable ordinal continuation ratio logistic analysis, the significant parameters were hypotension (odds ratio, OR = 1.9, 95% CI = 1.3–2.8, *p* = 0.001), tachycardia (OR = 1.8, 95% CI = 1.3–2.6, *p* < 0.001), Glasgow Coma Scale ≤8 (OR = 2.7, 95% CI = 1.9–3.7, *p* < 0.001), and traffic injury (OR = 1.8, 95% CI = 1.3–2.4, *p* < 0.001). The coefficients of these parameters were converted to item scores by choosing the lowest (0.57) as the smallest common division and rounding to the nearest 0.5 integer. The item scores ranged from 1 to 2, and total scores from 0 to 5 (Table 2). The general design and example scenario are provided in the Supplementary Materials S1.

**Figure 2:**
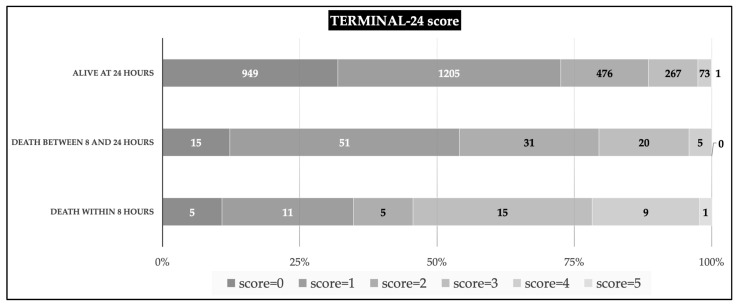
The distribution of TERMINAL-24 score by criterion-classified severity level; stacked bar charts with 0–100% bar for median/IQR (at 25%, 50%, and 75%); for each outcome group; the number in the bar chart represents the number of patients.

**Figure 3:**
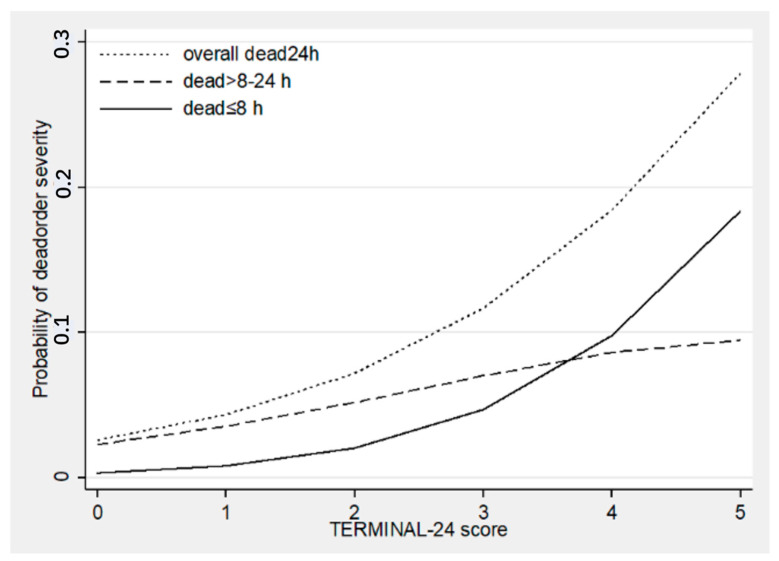
The discrimination of the probability of death based on TERMINAL-24 score. The dotted line represents the probability of overall death in 24 h, the dashed line represents the probability of death between 8 and 24 h, and the solid line represents the probability of death in under 8 h.

Applying a TERMINAL-24 score to all data, we found that the median and interquartile range of the scores among three groups: death in under 8 h, death between 8 and 24 h, and alive at 24 h were 3 (1,3), 1 (1–2), and 1 (0–2), respectively. The differences between scores and distribution across levels are illustrated as a stacked bar chart (Figure 2). The probability of death was observed as the exponential pattern at a score over 2 in the overall rate of death. The crossing between groups was observed at a score over 3, indicating the separation between the time of death under 8 h and that between 8–24 h (Figure 3). The Hosmer–Lemeshow goodness-of-fit test was used to assess the precision of the TERMINAL-24 score. The *p*-values for the groups of death under 8 h and between 8 and 24 h were 0.31 and 0.53, respectively. The indication was that the precision of the model was acceptable (0.1: lack of fit; almost 1: very fit).

**Figure 4:**
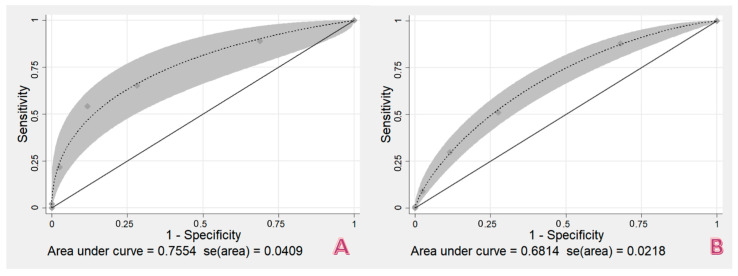
The area under ROC curve of TERMINAL-24 score in (**A**) death within 8 h and (**B**) death within 24 h.

**Figure 5:**
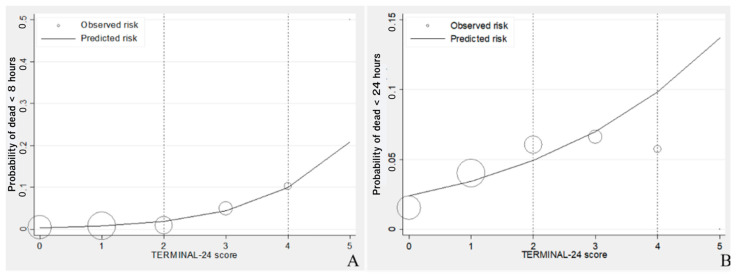
Risk curve of TERMINAL-24 score in (**A**) the death within 8 h group and (**B**) the death within 24 h group. The lines represent the predicted risks using TERMINAL-24 score. The circles represent the observed risks, and the size of circles represents the sample size.

The TERMINAL-24 score gave a poor to fair prediction for death of patients in the moderate group (under 24 h death) with an area under the ROC curve of 0.6814 (95% CI = 0.6387–0.7241). It predicted the severe group (death in under 8 h) more effectively with a greater area under the ROC curve of 0.7554 (95% CI = 0.6752–0.8356), as illustrated in Figure 4. The risk curve of within 8 h and within 24 h death demonstrated the correction of the TERMINAL-24 score in predicting the order of death, i.e., the predicted and observed risks increased in accordance with the TERMINAL-24 scores. Moreover, the predicted risk in the death within 8 h group was closer to the observed risk than that found in the group of 24 h death, as shown in Figure 5.

To ensure efficacy in clinical use, the sensitivity and specificity of the TERMINAL-24 score were calculated in the two groups, death under 8 h and between 8 and 24 h. The mortality rate was also calculated in association with the TERMINAL-24 score. It was indicated that at a score of 3, the mortality rate was higher than 10%. It was supported by a high specificity of 0.98 in both bands. Therefore, we suggested a TERMINAL-24 score of 3 as a mark for ICU admission. A TERMINAL-24 score of 4 or more showed a higher potential mortality rate (more than approximately 16%), indicating essential intervention by both an emergency doctor and a surgeon with the need for a specific treatment (Table 3).

We calculated a risk estimation validity of three levels of urgency classified by TERMINAL-24 score with the criteria of severity levels of the patients. The risk estimation validity indicated 85% exact correction, 11% overestimation, and 4% underestimation (Table 4). The likely ratio of a positive of a low-risk group (score 0–2) and a high-risk group (score 4–5) was 0.5 and 8.7, respectively (Table 5). The internal validation of the TERMINAL-24 score by the bootstrapping method was displayed as the mean optimism. The two groups death in under 8 h and death in under 24 h yielded a mean optimism of 0.0039 and 0.0033, respectively.

## 4. Discussion

Our study resulted in a novel informative continuous ratio ordinal outcome, which predicted the time of death of trauma patients who visited an emergency department during the first 24 h. This is important because in the majority of trauma patients, time has been spent in resuscitation in the first few hours before admission into hospital, thus their time in the emergency room was not longer than 8 h. Therefore, the focus of this study was the possibility of death in under 8 h and between 8 and 24 h. We also suggested that this ordinal outcome would be more effective than a binary outcome, specifically at the cutoff point of death in less than 24 h, as shown by the area under the ROC of 75% and 68% in under 8 h death and between 8 and 24 h death, respectively, while the binary outcome, dead or alive at 24 h, yielded an area under ROC of 70%. As an emergency physician, the death of multiple trauma patients should be classified as a continuous process, thus this continuous ratio ordinal outcome should be chosen.

We also included a systolic blood pressure parameter with only one cut-point, i.e., <90 mmHg, into our scoring system, while other systems suggested various cutoff points, e.g., MGAP [9] and GAP [10] use three cutoff points of >120, 60–120, and <60 mmHg, RTS [4] includes four cutoff points of 0, 1–49, 50–75, >89 mmHg, and NTS [21] employs five cutoff points of <70, 70–89, 90–109, 110–149, and >150 mmHg. We chose only one cutoff point in our study because a simple practical parameter would be more efficient in an emergency department where time taken to make decisions is crucial.

We derived a simple scoring system to predict 24 h mortality in major trauma adult patients for an emergency setting. Our scoring system would be more practicable than previous scoring systems, for example RTS and ISS. RTS is a score that calculates the probability of death for each patient [4], but it does not suggest any clinical judgment, while our score divided the patients into three levels of risk of early death. By using our score, we could manage the patients by the level of severity and thus prioritize patients on admission. The ISS score can be calculated after an investigation or treatment [5], whereas our score evaluates the patients at the initial state; thus, they are not comparable. Usefully, our “TERMINAL-24 score” does not require any laboratory or radiology results, in contrast to an EMTRAS score. It also classified the early death using the definitions described by the ATLS in an emergency room, time of death within 8 h, making the system suitable for emergency physicians who need to advise patients and their relatives, as well make decisions as to the best way forward with regard to surgery or other treatment. The TERMINAL-24 score has been designed to facilitate the planning of subsequent treatment, in contrast to other scoring systems whose purpose is to predict in-hospital death. In addition, those scoring systems monitor the time of death at 24 h, while TERMINAL-24 can be used to predict death within 8 h with a high level of precision. This is also advantageous for emergency doctors in Thailand as it enables the following of the trauma patients to subsequent shifts. There have been recent developments of new prediction models including machine learning [22], federated learning [23], mathematic algorithm [24], or system scoring. The last one was adopted for the performance of the TERMINAL-24 score. 

This TERMINAL-24 score could be used to predict early death by dividing the time of death into three levels; dead within 8 h, dead between 8 and 24 h, and alive after 24 h; therefore, it can be applied in any emergency settings. For example, if patients arrive at an ED that has insufficient resources, the TERMINAL-24 score could be used to predict the patients in the high-risk group of patients, enabling immediate referral to the trauma center. Furthermore, this score could identify the patients at a high risk of mortality within 8 h in the trauma center, facilitating the prioritization of the most severe patients for emergency intervention. We found that our “TERMINAL-24 score” predicted early death competently. This made the score practicable in overcrowding situations in the emergency room, which can commonly occur in a tertiary care hospital that serves many patients, not only those that have been referred but also walk-in patients and when there are cases of mass casualty. This scoring system can be used for prioritizing trauma patients for admittance to ICU or to a general ward, as well as in other settings of overcrowding in the emergency room. Although the internal validation of TERMINAL-24 yielded a satisfactory mean optimism level, our scoring system still requires other validations to establish its discriminative ability and precision in future studies. However, due to fewer parameters being required for the calculation of this score, it can be applied by any medical professional in their setting. Moreover, we are planning to validate this score with another dataset from a different setting in the future.

The limitation of this score is that although it can predict the overall mortality rate for each level of severity, it cannot define the time of death of an individual patient. The external validation should be done.

## 5. Conclusions

The TERMINAL-24 scoring system was competent for the prediction of early death in admissions to the Emergency Department, especially for the band death within 8 h, which would be appropriate in any emergency setting. Importantly, this scoring system does not require any laboratory or radiology results, with simple, solely practical parameters being sufficient to predict the probability of mortality. However, further evaluation of this score in different settings is needed before it can be routinely used in clinical practice.

## Figures and Tables

**Table 1:** Characteristics of patients with major trauma, criterion-classified into three levels of severity.

Patient Characteristics		Dead < 8 h	Dead 8–24 h	Alive at 24 h	*p*-Value *	Missing
		*n* = 46	*n* = 123	*n* = 3004		
		*n* (%)	*n* (%)	*n* (%)		*n* (%)
Demographics	Male	39 (84.8)	85 (69.1)	2179 (72.5)	0.362	0
	Age ≥ 50 years	13 (28.3)	53 (43.1)	1031 (34.3)	0.566	0
Vital signs	SBP < 90 mmHg	14 (30.4)	15 (12.3)	278 (9.3)	<0.001	25 (0.79)
	DBP < 60 mmHg	17 (37)	27 (22.1)	559 (18.8)	0.003	25 (0.79)
	PR ≥ 120/min	17 (37)	25 (20.5)	450 (15.1)	<0.001	18 (0.57)
	Abnormal RR (<10, >30/min)	22 (47.8)	46 (37.7)	722 (24.1)	<0.001	12 (0.38)
Mechanism of injury	Traffic	24 (52.2)	75 (61)	1432 (47.8)	0.026	9 (0.28)
	Fall	10 (21.7)	30 (24.4)	675 (22.5)	0.828	0
	Assault	7 (15.2)	27 (22)	426 (15)	0.103	0
Type of injury	Blunt	33 (71.7)	66 (55)	2058 (68.7)	0.111	11 (0.35)
	Penetrating	2 (4.3)	7 (5.8)	107 (3.6)	0.314	11 (0.35)
	Combination	3 (6.5)	15 (12.5)	373 (12.5)	0.352	11 (0.35)
	Other	8 (17.4)	32 (26.7)	458 (15.3)	0.018	11 (0.35)
Glasgow Coma Scale	Mild (14–15)	19 (41.3)	71 (58.2)	2103 (70.2)	<0.001	11 (0.35)
	Moderate (9–13)	1 (2.2)	12 (9.8)	321 (10.7)	0.165	11 (0.35)
	Severe (3–8)	26 (56.5)	39 (32)	570 (19)	<0.001	11 (0.35)
Transfer by	EMS	34 (74)	66 (53.7)	1751 (58.3)	0.309	217 (6.84)
	Referral	12 (26)	58 (47.2)	1213 (40.4)	0.557	0

* *p*-value from nonparametric test for trend; SBP: systolic blood pressure; DBP: diastolic blood pressure; PR: pulse rate; RR: respiratory rate; EMS: emergency medical service; mmHg: millimeters of mercury; min: minutes.

**Table 2:** Significant predictors of the three time bands of death (dead ≤ 8 h, dead 8–24 h, alive at 24 h) and assigned item score.

Predictors	Category	OR	95% CI	*p*-Value	Coefficient *	Score
Systolic blood pressure (mmHg)	<90 mmHg	1.88	1.28, 2.75	0.001	0.63	1
	≥90 mmHg	1	ref			0
Pulse (/min)	≥120 mmHg	1.84	1.32, 2.56	<0.001	0.61	1
	<120 mmHg	1	ref			0
Glasgow Coma Scale	Severe (3–8)	2.71	1.98, 3.70	<0.001	1.00	2
	Mild to moderate (9–15)	1	ref			0
Traffic injury	Yes	1.76	1.31, 2.38	<0.001	0.57	1
	No	1	ref			0

* Coefficients from multivariable continuation ratio logistic regression; OR: odds ratio; CI: confidence interval, ref: reference category; mmHg: millimeters of mercury; min: minutes. (TERMINAL-24 score = 1 * hypotension (SBP < 90) + 1 * tachycardia (HR ≥ 120) + 2 * coma (Glasgow coma scale < 9) + 1 * traffic injury).

**Table 3:** TERMINAL-24 score of three groups: death within 8 h, death between 8 and 24 h, and alive at 24 h. The sensitivity and specificity were calculated for the score in the following groups: death in under 8 h and death between 8 and 24 h. The mortality rate is shown in association with the TERMINAL-24 score.

TERMINAL-24 Score	Death within 8 h			Death between 8 and 24 h			Survival at 24 h	Mortality Rate (%)
	*n*	Sensitivity	Specificity	*n*	Sensitivity	Specificity	*n*	
0	5	0.89	0.32	15	0.88	0.32	949	2.06
1	11	0.65	0.73	51	0.46	0.73	1205	4.89
2	5	0.54	0.89	31	0.20	0.89	476	7.03
3	15	0.22	0.98	20	0.04	0.98	267	11.59
4	9	0.02	0.98	5	0.00	0.98	73	16.09
5	1	0.00	1.00	0	0.00	1.00	1	50.00

**Table 4:** Score-classified levels of urgency, criterion-classified levels of severity, and validity of risk estimation validity.

Score-Classified Urgency Levels		Score Range	Criterion-Classified Severity Levels			Risk Estimation Validity *		
			Alive at 24 h	Dead 8–24 h	Dead < 8 h	Over	Correct	Under
			*n* = 2971	*n* = 122	*n* = 46	(%)	(%)	(%)
Median (IQR)			1 (0,2)	1 (1,2)	3 (1,3)			
Low risk	*n* = 2748	0–2	2630	97	21	-	83.8	3.8
Moderate risk	*n* = 302	3	267	20	15	8.5	0.6	0.5
High risk	*n* = 89	4–5	74	5	10	2.5	0.3	-
					Total	11	84.7	4.3

* Percentage of total patients; h: hours.

**Table 5:** Score-classified levels of urgency, criterion-classified levels of severity, likelihood ratio of positive, 95% confidence interval, and *p*-value.

Score-Classified Urgency Levels		Score Range	Criterion-Classified Severity Levels			LHR+	95% CI	*p*-Value
			Alive at 24 h	Dead 8–24 h	Dead < 8 h			
			*n* = 2971	*n* = 122	*n* = 46			
Low risk	*n* = 2748	0–2	2630	97	21	0.5	0.5–1.0	0.007
Moderate risk	*n* = 302	3	267	20	15	3.6	1.9–4.7	<0.001
High risk	*n* = 89	4–5	74	5	10	8.7	4–13.5	<0.001

## Data Availability

The datasets used during the current study are available from the first author on reasonable request.

## Supplementary Materials

The following supporting information can be downloaded at: https://www.mdpi.com/article/10.3390/healthcare10030577/s1.
